# Numerical Simulation Study on Residual Stress and Strain in the Curing and Molding of HTPB Two-Stage Solid Propellant

**DOI:** 10.3390/polym18131588

**Published:** 2026-06-26

**Authors:** Jinpeng Chang, Chunguang Xu, Yingjun Dai

**Affiliations:** 1 School of Mechanical Engineering, Beijing Institute of Technology, Beijing 100081, China; xucg@bit.edu.cn; 2 Shanghai Aerospace Power Technology Research Institute, Shanghai 201109, China; daiyj1989@163.com

**Keywords:** HTPB two-stage solid propellant, curing and molding, residual stress and strain, modulus *m*, length-to-diameter ratio *n*

## Abstract

Understanding the curing and molding process of HTPB two-stage solid propellants and their stress and strain distributions is essential for the efficient manufacturing, long-term storage, safe transportation, and reliable operation of solid rocket motors. In this study, the residual stress and strain generated during the curing and molding of HTPB two-stage solid propellants were numerically investigated. The mechanisms responsible for residual stress and strain were analyzed, the relaxation modulus was characterized using a Prony series and the WLF time–temperature superposition equation, and the curing and cooling processes of a two-stage solid propellant grain were simulated. Furthermore, the effects of the modulus *m* and length-to-diameter ratio *n* on the residual stress and strain fields were investigated. The results show that at the end of the curing and cooling of the grains, there are high stress and strain zones on the sides close to the core mold and the shell. At the connection point between the first-stage and second-stage grains, due to the different materials, there is a sudden change in stress and strain. The curing stage accounts for 32.1% of the total residual stress and 32.6% of the total residual strain. As the modulus *m* increases, the overall stress and strain of the grain increase. As the length-to-diameter ratio *n* increases, the overall stress and strain of the grain decrease. This work provides a basis for the dimensional design of two-stage solid propellant grains and the selection of critical regions for structural safety evaluation.

## 1. Introduction

With the rapid development of aerospace technology and the increasing frequency of regional conflicts, solid rocket motors have become important propulsion systems for aerospace vehicles and weapon systems. Solid propellants are the power sources for various solid rocket engines. They are typically produced by casting a mixture of liquid fuel, oxidizer, and binder into the engine and curing it in a mold at a specific temperature [1,2]. The complete manufacturing cycle includes both curing and subsequent cooling [3,4,5]. During curing, the binder undergoes chemical crosslinking to form a three-dimensional network, transforming the propellant from a highly viscous fluid into a solid. During this stage, shrinkage of the binder system generates curing stress. The curing process is an exothermic chemical reaction, thus generating thermal stress [6,7,8,9]. During cooling, the cured propellant is cooled from the curing temperature to room temperature. During this process, significant thermal stress is generated due to the change in the temperature field [10,11,12]. Based on their binder systems, solid propellants can be classified as double-base, composite, or modified double-base propellants. Hydroxyl-terminated polybutadiene (HTPB) propellant is a composite propellant widely used in strategic and tactical missile motors. Residual stress and strain inevitably remain in the propellant after curing, and adequate mechanical integrity is essential for safe manufacturing, transportation, storage, and operation. Therefore, understanding the evolution of temperature, curing degree, stress, and strain during propellant curing is essential for the efficient manufacturing and safe transportation, storage, and operation of solid rocket motors.

Numerical simulation is currently the primary method used to evaluate residual stress and strain in cured solid propellants. In many studies, curing shrinkage stress and strain are not modeled directly; instead, their effects are represented empirically by an equivalent temperature change during the cooling analysis [13,14,15]. For example, for a curing temperature of 50 °C, the stress-free temperature may be assumed to be 58 °C, after which a thermomechanically coupled analysis is performed to calculate stress and strain during cooling. Lei et al. [16] established a finite element model based on Schapery’s one-dimensional nonlinear viscoelastic theory and proposed a viscoelastic constitutive model considering the temperature effect. Their results showed that material nonlinearity at elevated temperatures significantly affects the structural integrity of solid propellant grains. Du et al. [17] analyzed the cooling process using the transient thermal-structural coupling method. They identified the critical locations and the times at which they occurred during cooling and determined the maximum equivalent strain and extreme temperature. Cui et al. [18] used ABAQUS software to build a finite element model of a star-shaped propellant grain and then conducted a simulation study on the complete pressure curing process. Chu et al. [19] evaluated the time-temperature-related behavior of the structural integrity of solid propellant grains under cooling loads using the time-temperature equivalence principle, cumulative damage theory, and finite element software.

Current studies commonly use small-deformation linear viscoelastic constitutive models to analyze stress and strain distributions during propellant curing and molding [20,21,22,23]. Most previous studies have focused on grain deformation and stress-concentration locations under thermal loading during curing and molding [24,25,26], primarily for single-stage propellant grains. However, few studies have examined curing shrinkage stress and strain in two-stage solid propellants or the effects of the geometric parameters *m* and *n* on structural safety. In this study, a three-dimensional finite element model was developed to simulate the curing and molding of two-stage solid propellants. Stress and strain distributions during curing and cooling were analyzed, critical regions in the two-stage grain were identified, and the effects of the modulus *m* and length-to-diameter ratio *n* on grain stress and strain were evaluated. The results provide guidance for the dimensional design of two-stage solid propellant grains and the selection of critical regions for safety evaluation.

## 2. The Formation Mechanism of Residual Stress and Strain

### 2.1. Transient Heat Conduction

During the curing and molding process of the propellant, the heat conduction process can be regarded as a continuous medium. For a continuous propellant medium material, the differential equation for heat transfer during the curing process can be established [27,28], as follows:

(1) $$-\frac{\partial q_{i}}{\partial x_{i}}+Q-\rho c\frac{\partial \phi }{\partial t}=0$$

where ϕ is the temperature field, Q is the volumetric heat-generation rate, $q_{i}$ denotes the components of the heat-flux vector, ρ is the material density, c is the specific heat capacity, t is time.

According to Fourier’s law of heat conduction, the different components of the heat flux vector can be expressed in terms of the temperature gradient as:

(2) $$q_{i}=-\lambda_{ij}\frac{\partial \phi }{\partial x_{i}}$$

where $\lambda_{ij}$ is the component of the thermal conductivity tensor of the material in a certain spatial direction. In numerical simulation studies, it is generally assumed that solid propellant materials are isotropic materials. Therefore, thermal conductivity is assumed to be constant and identical in all directions.

Substituting Equation (2) into Equation (1) yields the three-dimensional heat conduction differential equation satisfied in the domain, which is:

(3) $$\frac{\partial }{\partial x_{i}}\left(\lambda_{ij}\frac{\partial \phi }{\partial x_{j}}\right)+Q-\rho c\frac{\partial \phi }{\partial t}=0$$

(4) $$Q=\rho H\alpha \frac{d\alpha }{dt}$$

where Hα is the heat of the curing reaction. Under normal circumstances, the thermophysical parameters and heat of formation of materials may both be functions of temperature. During curing, chemical reaction energy is converted into heat and acts as an internal heat source, thereby affecting the magnitude and distribution of temperature within the propellant grain. However, during the cooling process, there is no effect from the heat of formation.

### 2.2. Curing Reaction Kinetics

The curing reaction process is a complex reaction composed of several basic reactions. According to the law of mass action and the curing reaction mechanism, the curing reactions of materials can be classified into *n*-th order reaction, autocatalytic reaction, and Kamal reaction. Among them, the Kamal reaction is a composite reaction, which includes an *n*-th order reaction and an autocatalytic reaction [29,30,31,32]. The curing reaction of HTPB solid propellant belongs to an *n*-th order reaction, and its reaction equation is as follows:

(5) $$\frac{d\alpha }{dt}=k{\left(1-\alpha \right)}^{n}$$

where *k* is the rate constant expressed by the Arrhenius equation, and *n* is the reaction order. A characteristic of an *n*-th order reaction is that the reaction rate is the greatest at *t* = 0 and then gradually decreases.

The reaction rate constant *k* is a function of the reaction temperature *T* (absolute temperature) and follows the Arrhenius equation, which is expressed as:

(6) $$k=A\exp\left(-E/RT\right)$$

where *A* is the apparent pre-exponential factor, *E* is the apparent activation energy, and R is the universal gas constant.

### 2.3. Thermochemical Strain

During the curing process, the thermochemical strain generated by the propellant grain is composed of curing shrinkage strain and thermal strain [33,34]. Since the influence of temperature during the curing process is relatively small, the curing shrinkage strain plays a dominant role, which is expressed as:

(7) $$\varepsilon_{kl}^{\mathrm{ct}}=\varepsilon_{kl}^{c}+\varepsilon_{kl}^{t}$$

where $\varepsilon_{kl}^{\mathrm{ct}}$ is the thermal chemical strain, $\varepsilon_{kl}^{c}$ is the curing shrinkage strain, $\varepsilon_{kl}^{t}$ is the thermal strain.

During the curing reaction of the propellant, the overall volume shrinks as the distance between molecules decreases. A cubic element with a length, width, and height of *l*_1_, *l*_2_, and *l*_3_, respectively, is taken for study. When the volume of the selected element changes, the length changes in the three main directions are ∆*l*_1_, ∆*l*_2_, and ∆*l*_3_, respectively, and the volume of the element satisfies the following relationship:

(8) $$\Delta V=l_{1}\Delta l_{2}l_{3}+\Delta l_{1}l_{2}l_{3}+\Delta l_{1}\Delta l_{2}l_{3}+l_{1}l_{2}\Delta l_{3}+l_{1}\Delta l_{2}\Delta l_{3}+\Delta l_{1}l_{2}\Delta l_{3}+\Delta l_{1}\Delta l_{2}\Delta l_{3}$$

The rate of change in the volume of the selected element, ∆*v*, can be expressed by the following equation:

(9) $$\Delta v=\frac{\Delta V}{l_{1}l_{2}l_{3}}=\varepsilon_{1}+\varepsilon_{2}+\varepsilon_{3}+\varepsilon_{1}\varepsilon_{2}+\varepsilon_{1}\varepsilon_{3}+\varepsilon_{2}\varepsilon_{3}+\varepsilon_{1}\varepsilon_{2}\varepsilon_{3}$$

The curing shrinkage of the propellant begins with the curing reaction and stops after the reaction is completed. Therefore, only thermal strain occurs during the cooling process. In numerical simulation studies, solid propellant materials are usually regarded as isotropic [35], and the curing shrinkage strain in the three principal axis directions of the element is equal, expressed as:

(10) $$\varepsilon^{c}=\sqrt[{3}]{1+\Delta v}-1$$

During the curing process of the propellant, the relationship between the volume change rate of the propellant grain and the total volume change *V*_sh_ during the curing stage and the curing degree is as follows:

(11) $$\Delta v=\Delta \alpha \cdot V_{\mathrm{sh}}$$

Then the curing shrinkage strain *ε*_c_ of the propellant grain is:

(12) $$\varepsilon^{c}=\sqrt[{3}]{1+\Delta \alpha \cdot V_{\mathrm{sh}}}-1$$

The curing and molding process includes the curing process and the cooling process, and their thermal strains can all be expressed as:

(13) $$\varepsilon^{t}=\alpha \Delta T$$

where *α* is the coefficient of thermal expansion of the propellant material.

### 2.4. Constitutive Model

Due to the extremely long curing process of solid propellants, the curing process is regarded as an elastic deformation stage. The elastic constitutive relations of the curing stage of the propellant are [36,37]:

(14) $$\sigma_{ij}\left(t,\alpha \right)=C_{ijkl}\left(\alpha \right){\hat{\varepsilon }}_{kl}\left(t\right)$$

From the assumption of isotropy in material mechanics, the following forms can be derived:

(15) $$\sigma_{ij}=\lambda \sigma_{kk}\delta_{ij}+2\mu \varepsilon_{ij}$$

where

(16) $$\left\{\begin{array}{l} \lambda =K-\frac{2\mu }{3}\\ \mu =G \end{array}\right.$$

where λ and μ are the Lame constants in the linear elastic theory of materials.

From Equations (14) and (15), the linear elastic constitutive equation for the material curing process can be derived as:

(17) $$\sigma_{ij}\left(t,\alpha \right)=\frac{E\left(\alpha \right)}{1+\nu }\left(\varepsilon_{ij}\left(t\right)+\frac{\nu }{1-2\nu }\delta_{ij}\varepsilon_{kk}\left(t\right)\right)$$

The propellant material in the cooling process is a viscoelastic material. When studying the linear viscoelastic problems of the material, the Boltzmann superposition principle is generally applied, which means that the sum of the individual effects of multiple causes is the total effect of their action. Therefore, the current stress depends on the superimposed effect of multiple continuous strain loads in the past. The stress relaxation constitutive relation can be expressed by the Stieltjes integral as [38,39,40,41]:

(18) $$\sigma_{ij}\left(t\right)=\int_{0}^{\infty }\left[\varepsilon_{kl}\left(t-s\right)-\delta_{kl}\alpha_{kl}T\left(t-s\right)\right]dG_{ijkl}\left(s\right)$$

where $G_{ijkl}$ is the relaxation function, $\alpha_{kl}$ is the coefficient of thermal expansion, T is the relative temperature change, $\delta_{kl}$ is 1 (when *k* = l), and $\delta_{kl}$ is 0 (when *k* ≠ l).

Suppose that $G_{ijkl}$ and its first-order partial derivative with respect to time are continuous on the interval, then Equation (18) can be rewritten as:

(19) $$\sigma_{ij}\left(t\right)=G_{ijkl}\left(0\right)\left[\varepsilon_{kl}\left(t\right)-\delta_{kl}\alpha_{kl}T\left(t\right)\right]+\int_{0}^{t}\left[\varepsilon_{kl}\left(t-s\right)-\delta_{kl}\alpha_{kl}T\left(t-s\right)\right]\frac{dG_{ijkl}\left(s\right)}{d\tau }ds$$

Solid propellants are generally regarded as isotropic materials in simulation studies. The material relaxation function does not change with direction and only has two independent coefficients, as follows:

(20) $$G_{ijkl}\left(t\right)=\frac{1}{3}\left[3K\left(t\right)-2G\left(t\right)\right]\delta_{ij}\delta_{kl}+G\left(t\right)\left(\delta_{ik}\delta_{jl}+\delta_{il}\delta_{jk}\right)$$

where $K\left(t\right)$ is the volume relaxation modulus, $G\left(t\right)$ is the shear relaxation modulus.

Assuming that Poisson’s ratio does not change over time, the relationship among the volume relaxation modulus, shear relaxation modulus, and tensile relaxation modulus is:

(21) E(t)=3K(t)(1−2ν)

(22) $$E\left(t\right)=2G\left(t\right)\left(1+\nu \right)$$

Then Equation (19) can be rewritten as:

(23) $$\sigma \left(t\right)=D\left[E\left(0\right)\left[\varepsilon \left(t\right)-\varepsilon_{T}\left(t\right)\right]\right]+D\int_{0}^{t}\left[\varepsilon \left(t-s\right)-\varepsilon_{T}\left(t-s\right)\right]\frac{dE\left(s\right)}{ds}ds$$

where

(24) $$\sigma ={\left[\sigma_{11}\;\sigma_{22}\;\sigma_{33}\;\tau_{12}\;\tau_{23}\;\tau_{31}\right]}^{T}$$

(25) $$\varepsilon ={\left[\varepsilon_{11}\;\varepsilon_{22}\;\varepsilon_{33}\;\gamma_{12}\;\gamma_{23}\;\gamma_{31}\right]}^{T}$$

(26) $$\varepsilon_{T}=\alpha_{T}T{\left[\begin{array}{cccccc} 1 & 1 & 1 & 0 & 0 & 0 \end{array}\right]}^{T}$$

(27) $$D=\frac{1-\nu }{\left(1+\nu \right)\;\left(1-2\nu \right)}\left[\begin{array}{cccccc} 1 & \frac{\nu }{1-\nu } & \frac{\nu }{1-\nu } & 0 & 0 & 0\\ \frac{\nu }{1-\nu } & 1 & \frac{\nu }{1-\nu } & 0 & 0 & 0\\ \frac{\nu }{1-\nu } & \frac{\nu }{1-\nu } & 1 & 0 & 0 & 0\\ 0 & 0 & 0 & \frac{1-2\nu }{2\left(1-\nu \right)} & 0 & 0\\ 0 & 0 & 0 & 0 & \frac{1-2\nu }{2\left(1-\nu \right)} & 0\\ 0 & 0 & 0 & 0 & 0 & \frac{1-2\nu }{2\left(1-\nu \right)} \end{array}\right]$$

## 3. Simulation Model

**Figure 1:**
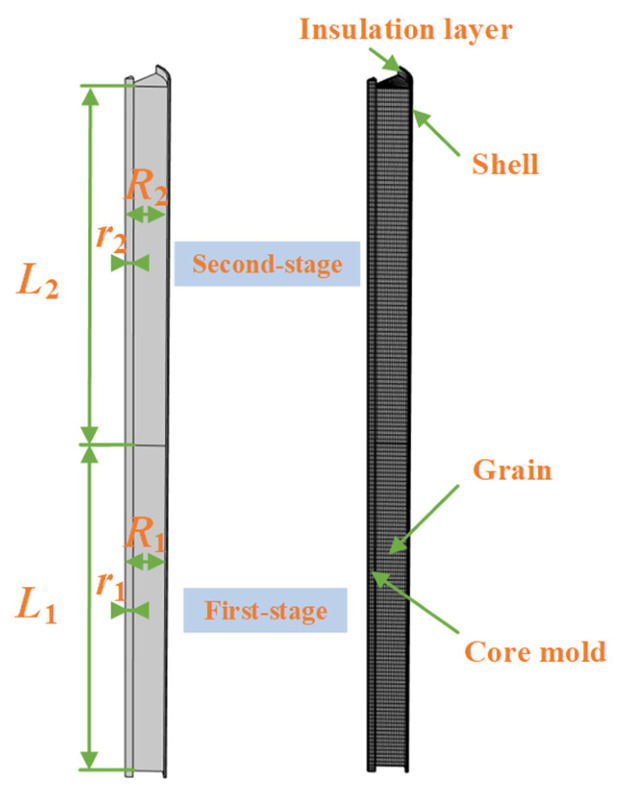
Simulation model and meshing.

Taking the two-material two-stage cylindrical tube type propellant structure as the research object, the solid rocket motor is composed of four parts: the shell, the insulation layer, the propellant grain, and the core mold. The propellant grain is divided into the first-stage grain and the second-stage grain. Perfect bonding was assumed between the first-stage and second-stage propellant grains, and the interface was modeled using continuity boundary conditions. Due to the symmetry of the propellant structure, a 1/8 finite element simulation model was established as shown in Figure 1. During the simulation study, the dimensions of the first- and second-stage propellant grains are kept consistent, that is, the height of the propellant grain *L* = 2*L*_1_ = 2*L*_2_ = 1000 mm, the outer radius of the propellant grain *R* = *R*_1_ = *R*_2_ = 50 mm, and the inner radius *r* = *r*_1_ = *r*_2_ = 10 mm. The thickness of the insulation layer is 2 mm, and the shell thickness is 1 mm. The modulus of the propellant grain is defined as *m* = *R*/*r* = 5, and the length-to-diameter ratio is *n* = *L*/2*D* = 5, where *D* is the outer diameter of the propellant grain.

The finite element model was established in COMSOL Multiphysics 6.3. Hexahedral elements were used for the propellant grain, insulation layer, and shell, while tetrahedral elements were employed for the core mold. Local mesh refinement was applied in regions with large stress gradients, including the propellant-insulation interface and the interface between the first-stage and second-stage propellants. To verify the mesh independence and ensure the efficiency and accuracy of the calculation, three different mesh sizes were used for simulation in this paper. The maximum stress data after the curing and cooling of the propellant grain are extracted, and the results are shown in Table 1. Using a medium mesh size can ensure the calculation accuracy. Based on the mesh independence analysis, the medium mesh containing 38,720 elements and 44,836 nodes was adopted for all subsequent simulations.

### 3.1. Material Parameters

The rocket engine shell is made of 30Cr3SiNiMo alloy steel, the solid propellant charge is HTPB propellant, and the core mold material is hard aluminum 2A12. Its curing temperature is 60 °C and the curing period is 8 days. The cured rocket engine was placed in an environment of 20 °C to cool down to room temperature naturally, with a convective heat transfer coefficient of 15W/(m^2^·K). Other material parameters are shown in Table 2.

Both stages were composed of HTPB propellants with the same binder type and curing conditions. Therefore, it is assumed that the curing kinetics parameters of the two stages are consistent. The curing kinetics parameters of the HTPB propellant during the curing process are listed in Table 3.

The curing process of the propellant can be regarded as a linear elastic deformation process. Therefore, the instantaneous elastic modulus of the propellant can be expressed as $E(t)=E_{\infty }\alpha (t)$, where $E_{\infty }$ is the elastic modulus at full curing, taken as the equilibrium modulus of the relaxation modulus at the beginning of the cooling stage. This is because the curing process is very long, and the material is always in a state close to relaxation. The total volume change *V*_sh_ of the propellant grain during the curing process is 0.5%.

### 3.2. Relaxation Modulus

**Figure 2:**
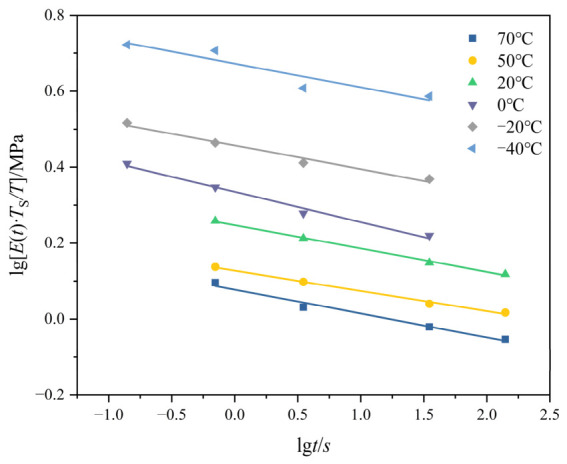
Lg[*E*(*t*)·*T*_s_/*T*]-lg(*t*) relationship.

When determining the material relaxation modulus of the first-stage propellant charge, the dumbbell-shaped specimens were tensile tested at temperatures of 70 °C, 50 °C, 20 °C, 5 °C, −20 °C, and −40 °C, with tensile speeds of 500 mm/min, 100 mm/min, 20 mm/min, 2 mm/min, and 0.5 mm/min. The reference temperature *T*_s_ was 20 °C. Following the method proposed by Yu [42], the ratio of tensile strength to elongation was used as an approximate indicator of the modulus at the corresponding loading rate. The corresponding lg*t* value was calculated based on the tensile speed. The logarithm of the modulus value at different temperatures and tensile speeds converted to the modulus value at a certain reference temperature, that is, lg[*E*(*t*)·*T*_s_/*T*], was plotted as a lg[*E*(*t*)·*T*_s_/*T*]-lg*t* relationship graph as shown in Figure 2.

At the reference temperature of 20 °C, the lg[*E*(*t*)·*T*_s_/*T*]-lg*t* relationship curves at various temperatures were plotted using the translation graph method to obtain the temperature transfer factor lg*α_T_*, as shown in Table 4.

**Figure 3:**
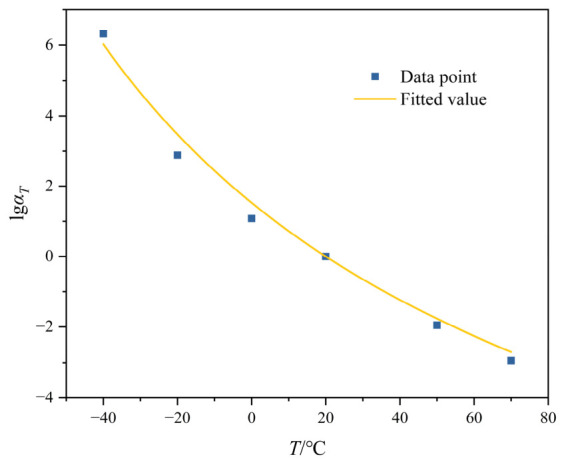
Lg*α_T_*-*T* relationship.

The curve graph of the horizontal displacement factor lg*α_T_* was obtained by fitting the lg*α_T_*-*T* with the WLF equation through the above table, as shown in Figure 3. The temperature transfer factor:

(28) $$\mathrm{lg}\alpha_{T}=\frac{-18.28\left(T-20\right)}{256.97+\left(T-20\right)}$$

**Figure 4:**
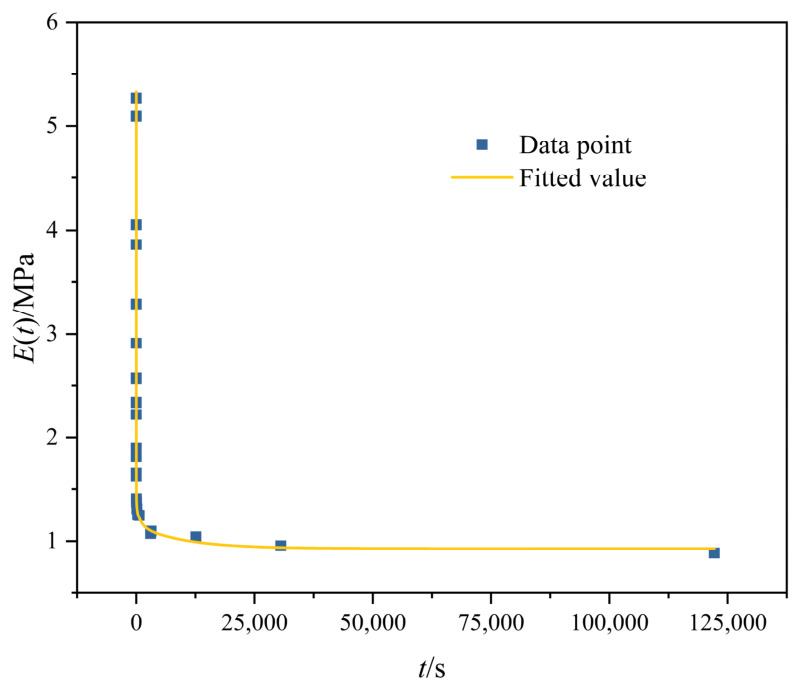
Relaxation modulus curve.

In the plane rectangular coordinate system, with *t*/*α_T_* as the abscissa and *E*(*t*)·*T*_s_/*T* as the ordinate, a scatter plot is drawn and fitted into the form of $E\left(t\right)=E_{e}+\overset{n}{\underset{i=1}{\sum }}E_{i}\exp\left(-t/\tau_{i}\right)$. The curve is shown in Figure 4. Among them, the equilibrium modulus $E_{e}$ = 0.935 MPa; $\tau_{i}$ = 10*^i^*^−7^ (*i* = 1, 11); see Table 5.

The method for obtaining the relaxation modulus of the second-stage propellant grain is the same as that of the first-stage propellant grain. Its relaxation modulus can be expressed by the Prony series as shown in Table 6.

The equilibrium modulus $E_{e}$ = 1.456 MPa, $\tau_{i}$ = 10*^i^*^−6^ (*i* = 1, 9). The temperature transfer factor can be expressed as:

(29) $$\mathrm{lg}\alpha_{T}=\frac{-12.95\left(T-20\right)}{186.25+\left(T-20\right)}$$

### 3.3. Model Validation

**Figure 5:**
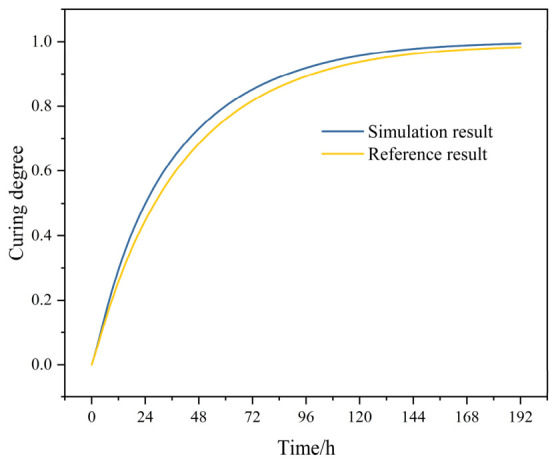
Comparison of the curing degree between simulation results and results from reference [43].

To verify the accuracy of the simulation model adopted in this paper, the curing process results of the finite element simulation were compared with the experimental results obtained by Lee et al. [43]. The numerical solution method of curing kinetics was exactly the same as that in reference [43]. Figure 5 shows the comparison of the curing degree curves from reference [43]. The simulated curing degree curve agrees well with the experimental data, indicating that the model adequately captures the curing behavior of the propellant.

## 4. Simulation Results and Analysis

### 4.1. Curing Process Temperature and Curing Degree

**Figure 6:**
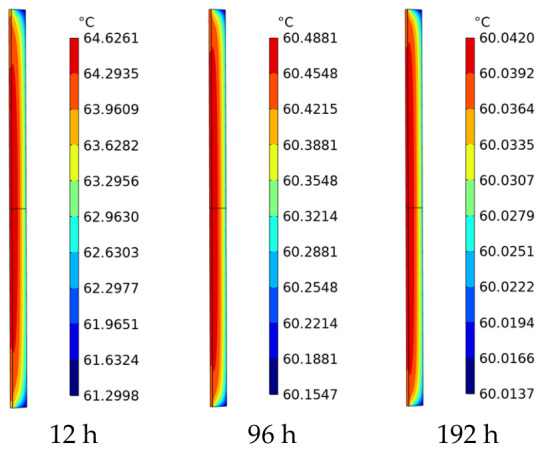
The temperature during the curing.

Temperature contours of the HTPB propellant grain at the early, intermediate, and final stages of curing are shown in Figure 6. During each period of the curing process, the temperature on the side of the grain close to the core mold is higher than that on the side close to the shell. This is because the curing reaction is an exothermic reaction, and the side close to the shell has a larger heat dissipation area. At 12 h of curing, the maximum temperature of the grain is obviously higher than that in the middle and later stages of curing. When curing for 96 h and 192 h, the temperature of the entire grain tends to be consistent, approaching the ambient temperature of 60 °C. This indicates that the curing reaction rate is faster in the early stage, and thus the heat release rate is also faster, resulting in a higher temperature. In the middle and later stages of curing, the curing reaction rate is slower, leading to a lower temperature. This shows that the heat release of the curing reaction is mainly concentrated in the early stage of curing.

**Figure 7:**
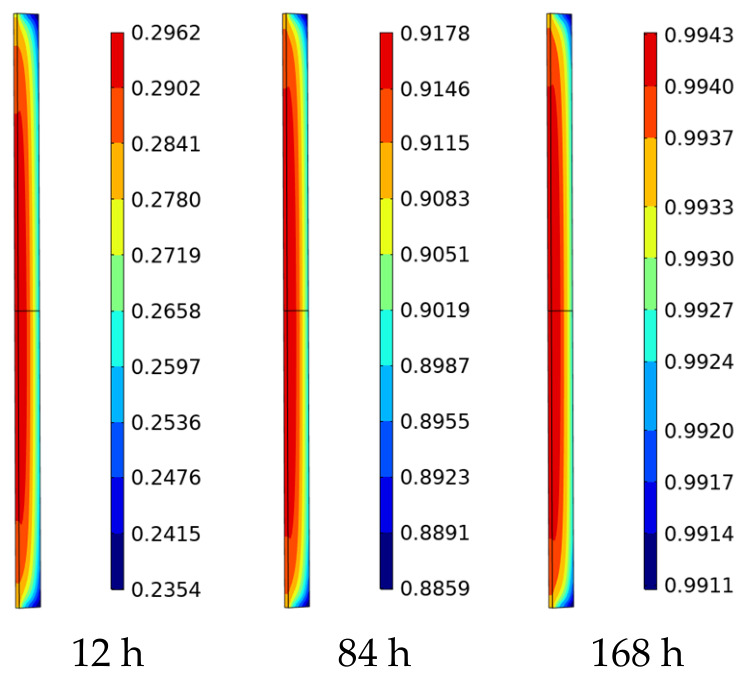
Curing degree during the curing.

The curing degree contour plots of the propellant grain at different curing periods are shown in Figure 7. The curing degree distribution at different curing periods is similar to the temperature distribution. The curing degree on the side of the grain close to the core mold is higher than that on the side close to the shell, which is obviously due to the influence of temperature on the curing rate. The higher the temperature, the faster the curing reaction rate. Moreover, the curing degree has reached about 0.9 at 96 h of curing, indicating that the curing reaction rate is extremely slow in the later stage of curing. This is because the curing reaction rate is influenced not only by temperature but also by the curing degree. As the curing degree increases, the curing reaction rate gradually decreases. At the end of curing, the curing degree of the entire grain tends to be uniform, reaching above 0.991.

**Figure 8:**
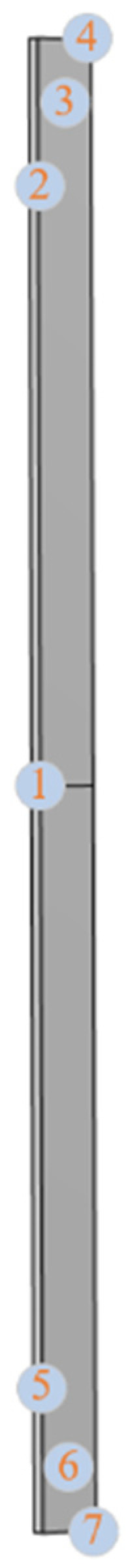
Selected points.

**Figure 9:**
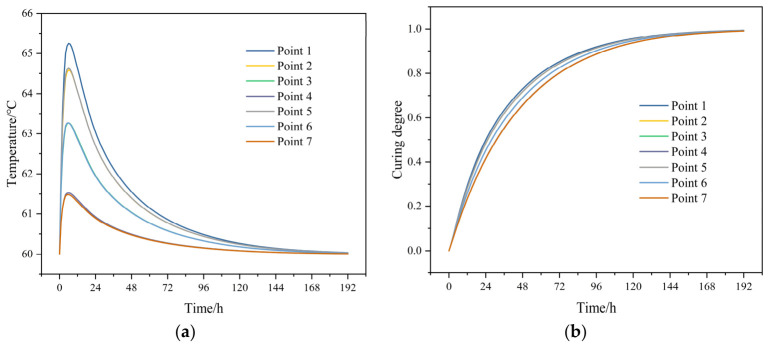
Variation in selected point temperature and curing degree with curing time: (**a**) temperature; (**b**) curing degree.

As shown in Figure 8, seven points in different regions with significant variations in temperature and curing degree were selected for analysis. The selected points were distributed in representative regions at different distances from the core mold and the shell, as well as at geometrically symmetric locations, to investigate the spatial distribution of temperature and degree of cure during the curing process. The changes in temperature and curing degree of these selected points over the curing time are presented in Figure 9. From Figure 9a, it can be observed that the temperature in different regions of the propellant grain initially increases and then decreases, reaching the maximum value around 6 h of curing time. The maximum temperature of the propellant grain reaches 65.2 °C, and the temperature difference between adjacent regions also reaches its maximum, with a maximum difference of 3.7 °C. After 168 h of curing, the overall temperature of the propellant tends to be uniform, approximately 60 °C. From Figure 9b, it can be seen that the curing reaction rate of each point reaches its maximum at the beginning of the curing reaction and then gradually decreases, which reflects the characteristics of the *N*-th order curing kinetics. After 180 h, the curing degree of each point tends to stabilize. During the period from 12 to 132 h of curing, the curing degree of points 1, 2, and 5, which are closer to the core mold, is significantly higher than that of other points. At 48 h of curing, the maximum difference in curing degree between different points exists, approximately 0.073. The temperature and curing degree curves of points 2, 3, and 4 correspond to those of points 5, 6, and 7, respectively, which reflects the symmetry of the selected points. This is because the curing kinetics parameters of the first and second stages of the propellant grain are the same, with only a minor difference in thermal conductivity that can be ignored.

The curing reaction of the propellant is essentially a process of heat release from a chemical reaction. The temperature peak results from the competition between the heat released by the curing reaction and the heat dissipated to the surroundings. In the early stage of curing, the reaction rate predicted by the *n*-th order curing kinetics model is relatively high, leading to rapid heat generation. As the curing degree increases, the reaction rate gradually decreases and heat dissipation becomes dominant, causing the temperature to decrease after approximately 6 h. In the early stage of curing, the overall curing degree is not high, and the temperature differences between different points lead to different curing reaction rates in different regions of the propellant grain. Therefore, in the early stage of curing, there are significant temperature and curing degree differences in the propellant grain. In the middle and later stages of curing, the curing degree of the propellant grain has reached a high level. The continuous formation of the curing crosslinking network hinders the curing process of the uncured parts, so the corresponding reaction rate is very slow. The heat generated by the curing reaction of the propellant and the heat dissipation from the heat exchange with the external environment are basically balanced, so the overall temperature tends to be uniform.

### 4.2. Residual Stress and Strain

**Figure 10:**
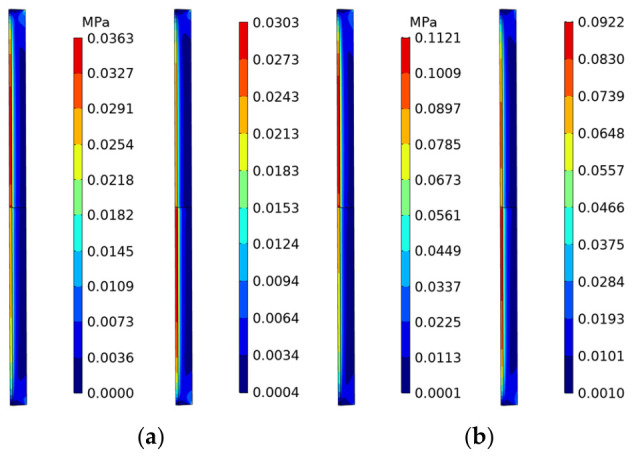
Residual stress and strain: (**a**) at the end of the curing; (**b**) at the end of the cooling.

**Figure 11:**
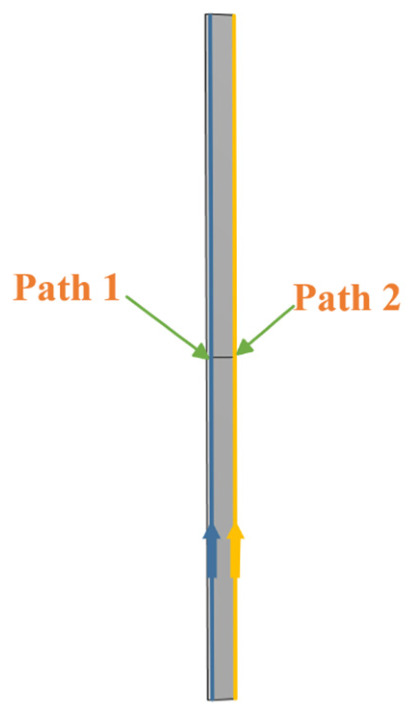
Selected path.

The residual stress and strain are expressed in terms of equivalent stress and strain, as shown in Figure 10. The stress and strain distribution at the end of the curing process and the cooling process are basically the same. There are high stress and strain zones on both sides of the propellant grain, close to the core mold and the side close to the shell. The high stress zone on the side of the propellant grain close to the core mold is on the second-stage propellant grain, while the high strain zone is on the first-stage propellant grain. The high stress and strain zones on the side of the propellant grain close to the shell are at the connection points between the ends of the propellant grain and the insulation layer. Due to the higher temperature and curing degree gradients on the core mold side, greater strain is generated. The modulus of the first-stage propellant grain is smaller and thus more prone to high strain, while the modulus of the second-stage propellant grain is larger, resulting in greater stress. Stress and strain concentration occur at the connection points between the ends of the propellant grain and the insulation layer due to the constraint of the shell. Therefore, one path is selected on each side for analysis, as shown in Figure 11.

**Figure 12:**
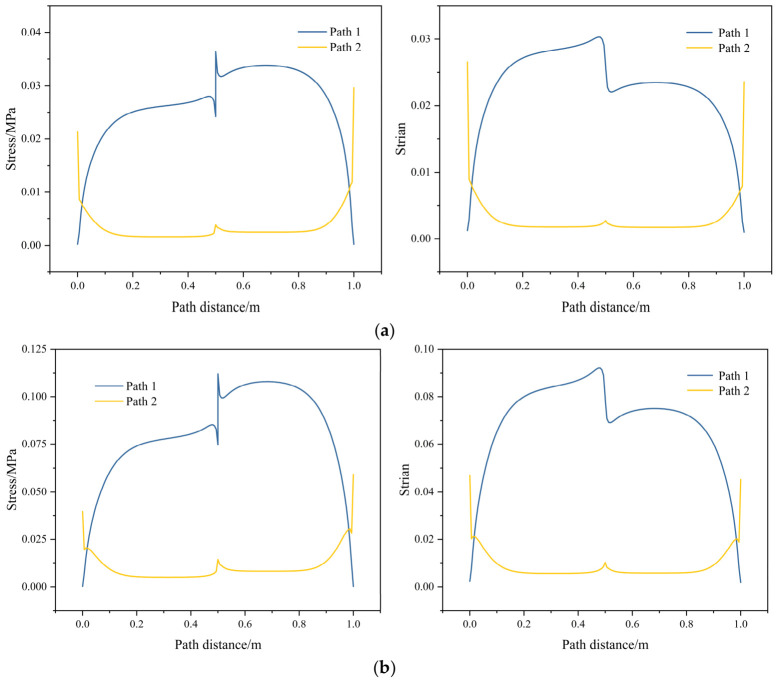
Variation in stress and strain with the selected path: (**a**) at the end of the curing; (**b**) at the end of the cooling.

The variation in stress and strain along different paths is shown in Figure 12. The maximum values of stress and strain occur at the junction of the first- and second-stage propellant grains in path 1, where a sudden change in stress and strain occurs due to the difference in material. After curing, the maximum stress is 0.036 MPa, and the maximum strain is 0.030. After cooling, the maximum stress is 0.112 MPa, and the maximum strain is 0.092. The proportion of residual stress generated during the curing process is 32.1%, and the proportion of residual strain is 32.6%. Path 1 mainly considers the deformation and cracking hazards of the propellant grains, while path 2 mainly considers the debonding hazards between the propellant grains and the insulation layer. Therefore, the maximum values on both paths need to be considered separately. In path 2, there is a significant concentration of stress and strain at the connection points between the propellant grains and the insulation layer at both ends, while the stress and strain are relatively small at other locations. After curing and cooling, the maximum stress is 0.059 MPa, and the maximum strain is 0.047. Although the maximum residual stress is considerably lower than the reported tensile strength of HTPB propellants, it may superimpose with subsequent service loads and therefore should not be neglected in structural integrity assessments.

### 4.3. The Influence of Modulus m

The main dimensions of a solid propellant grain during design include the inner and outer diameters and height, while the modulus *m* and the length-to-diameter ratio *n* define the relationship among these three. To study the influence of the modulus *m* on the curing process, the length-to-diameter ratio *n* was set to 5, and the modulus *m* was varied only by changing the inner radius. The cases of *m* = 2, 3, 4, and 5 were investigated.

**Figure 13:**
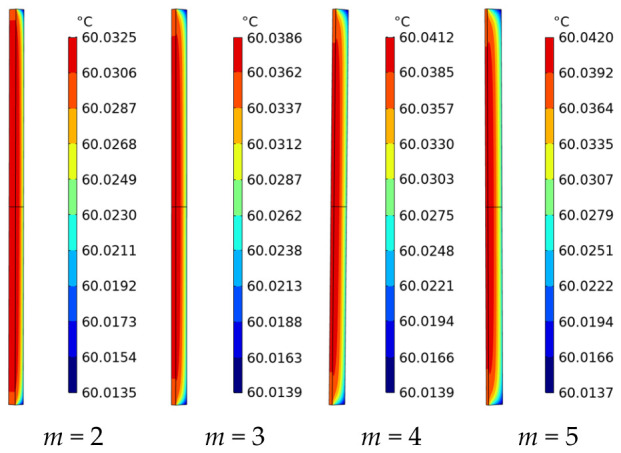
Temperature at the end of curing for propellant grains with different moduli *m*.

**Figure 14:**
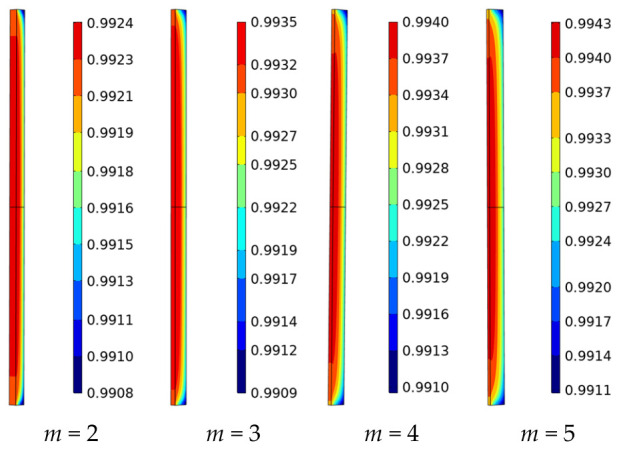
Curing degree at the end of curing for propellant grains with different moduli *m*.

As can be seen from Figure 13 and Figure 14, as the modulus *m* increases, the temperature and degree of curing at the end of the curing process of the grain increase, and the temperature difference and curing degree difference between the side close to the core mold and the side close to the shell also become larger. This is because, as the modulus *m* increases, the grain becomes thicker, and the increase in propellant mass leads to more heat generated by the curing reaction. However, the heat exchange area on the core mold side decreases, making it more difficult for the generated heat to exchange with the outside, resulting in a temperature rise. Correspondingly, the curing reaction rate also increases, ultimately leading to an increase in the curing degree.

**Figure 15:**
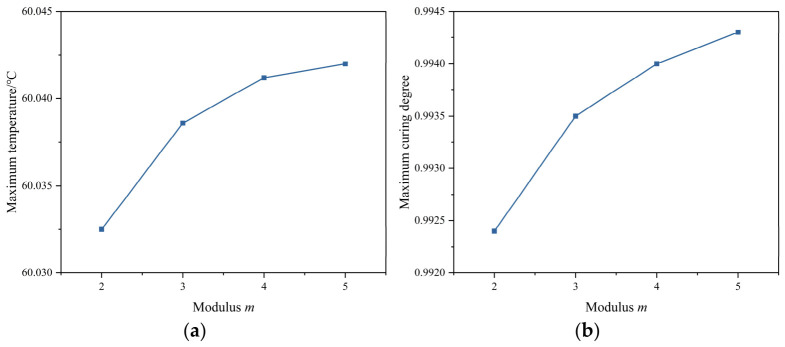
Variation in the maximum temperature and curing degree at the end of curing with modulus *m*: (**a**) maximum temperature; (**b**) maximum curing degree.

Further analysis of the maximum values of temperature and curing degree at the end of the curing process as a function of modulus *m* is shown in Figure 15. As modulus *m* increases, both the maximum temperature and the maximum degree of curing increase, but the rate of increase gradually decreases. This is because as modulus *m* increases by 1 each time, the increase in dosage of propellant is continuously decreasing, thus the temperature rise also becomes smaller, and correspondingly, the change in curing degree decreases.

**Figure 16:**
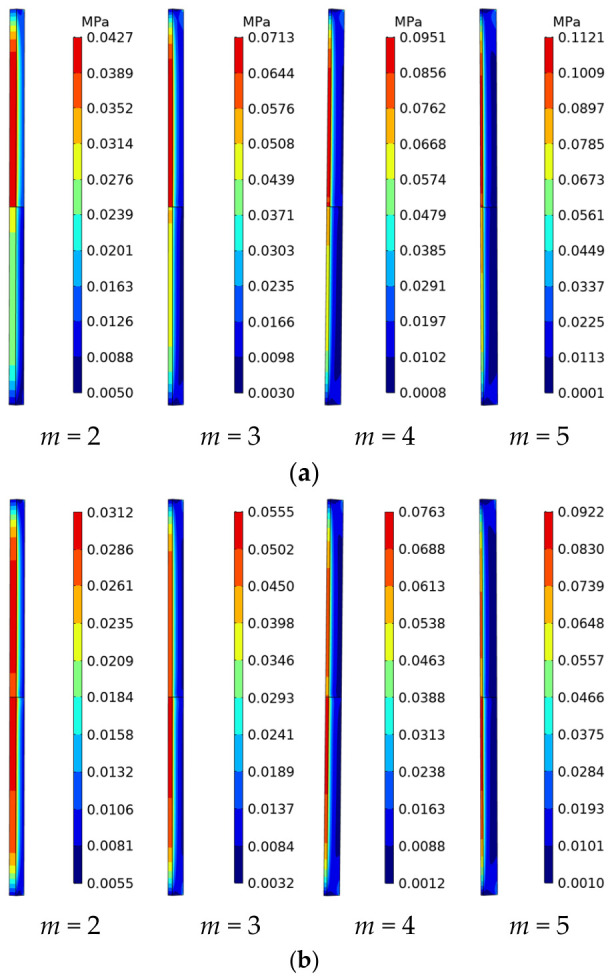
Stress and strain at the end of cooling for propellant grains with different moduli *m*: (**a**) stress; (**b**) strain.

As shown in Figure 16, the distribution of stress and strain at the end of cooling for propellant grains with different modulus *m* is similar. The high stress and strain areas are located on the sides of the grain close to the core and the shell. As the modulus *m* increases, the overall stress and strain of the grain increase because the higher temperature and curing degree gradients result in greater thermal strain and curing shrinkage strain in the grain.

**Figure 17:**
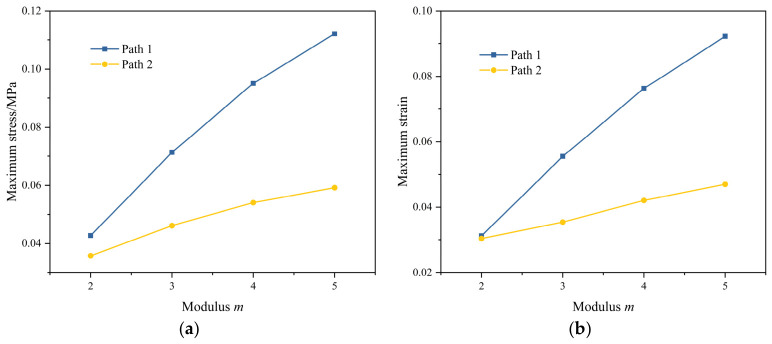
Variation in the maximum stress and strain along different paths with the modulus *m*: (**a**) maximum stress; (**b**) maximum strain.

The maximum values of stress and strain along two paths were extracted, respectively, and their variations with the modulus *m* are shown in Figure 17. The maximum values of stress and strain increase with the increase in modulus *m*, but the magnitudes of change along the two paths are different. Since the temperature and curing degree variations along path 1 are greater than those along path 2, the magnitude of change along path 1 is larger. For every increase of 1 in modulus *m*, the maximum stress increases by approximately 0.028 MPa, and the maximum strain increases by approximately 0.021. In contrast, the change along path 2 is relatively gentle, with the maximum stress increasing by approximately 0.008 MPa and the maximum strain increasing by approximately 0.007 for every increase of 1 in modulus *m*.

### 4.4. The Influence of the Length-to-Diameter Ratio n

**Figure 18:**
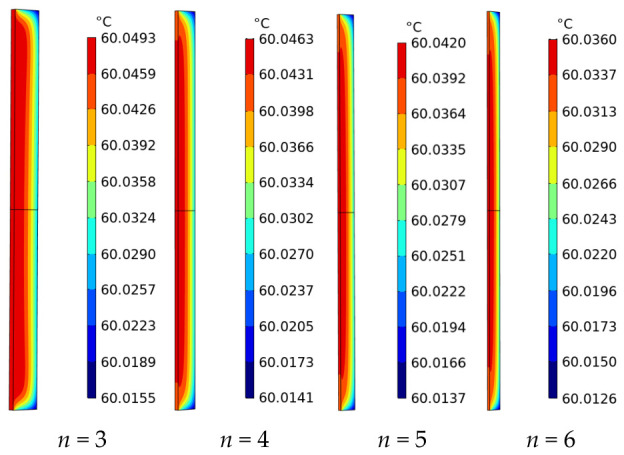
Temperature at the end of curing for propellant grain with different length-to-diameter ratios *n*.

**Figure 19:**
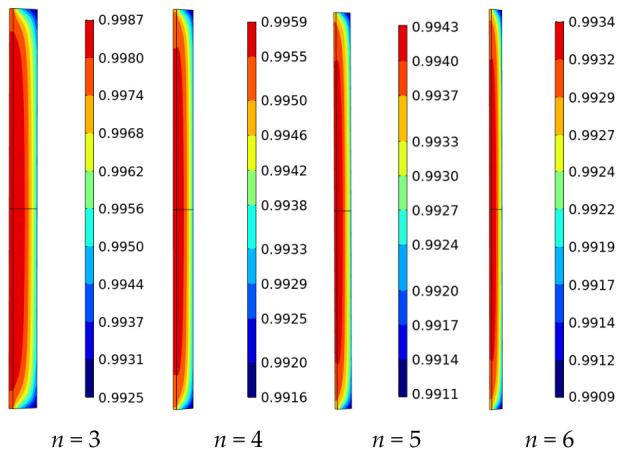
Curing degree at the end of curing for propellant grains with different length-to-diameter ratios *n*.

To study the influence of the length-to-diameter ratio *n* on the curing and molding process, the modulus *m* was set to 5, and the length-to-diameter ratio *n* varied only by changing the outer diameter. The cases of *n* = 3, 4, 5, and 6 were investigated. As can be seen from Figure 18 and Figure 19, as the length-to-diameter ratio *n* increases, the temperature and curing degree at the end of the curing process of the propellant grain decrease, and the temperature difference and curing degree difference between the side close to the core mold and the side close to the shell of the propellant grain tend to be consistent. This is because, as the length-to-diameter ratio *n* increases, the propellant grain becomes thinner, and the corresponding propellant quantity decreases, resulting in less heat generated. Enhanced heat dissipation lowers the internal temperature and curing rate, ultimately resulting in a lower overall curing degree.

**Figure 20:**
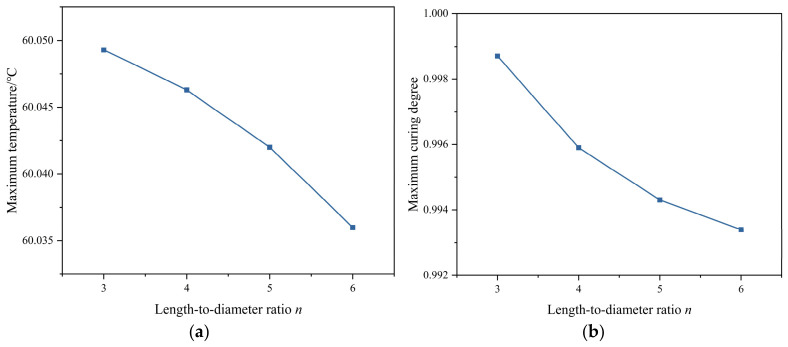
Variation in the maximum temperature and curing degree at the end of curing with the length-to-diameter ratio *n*; (**a**) maximum temperature; (**b**) maximum curing degree.

Further analysis of the variation in the maximum temperature and curing degree at the end of curing with the length-to-diameter ratio *n* is shown in Figure 20. As the length-to-diameter ratio *n* increases, both the maximum temperature and the maximum degree of curing decrease. The amplitude of the maximum temperature change gradually increases, whereas the amplitude of the maximum curing degree change gradually decreases.

**Figure 21:**
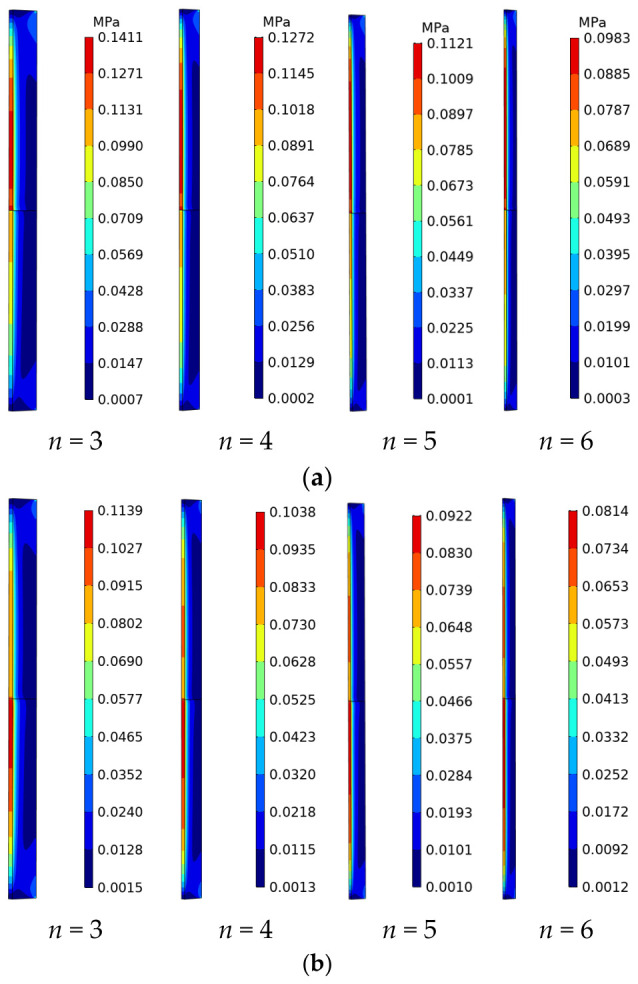
Stress and strain at the end of cooling for propellant grains with different length-to-diameter ratios *n*: (**a**) stress; (**b**) strain.

As shown in Figure 21, the distribution of stress and strain at the end of cooling for propellant grains with different length-to-diameter ratios *n* is similar. The high stress and strain regions are located on the sides of the grain close to the core and the shell. As the length-to-diameter ratio *n* increases, the overall stress and strain of the grain decrease. This is due to the smaller temperature and curing degree gradients, which result in smaller thermal strain and curing shrinkage strain in the grain.

**Figure 22:**
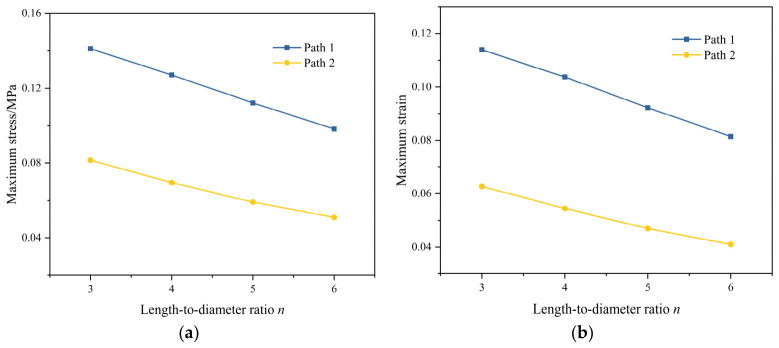
Variation in the maximum stress and strain along different paths with the length-to-diameter ratio *n*: (**a**) maximum stress; (**b**) maximum strain.

The maximum values of stress and strain along two paths were extracted, respectively, and their variations with the length-to-diameter ratio *n* are shown in Figure 22. The maximum values of stress and strain decrease with the increase in the length-to-diameter ratio *n*. For path 1, the maximum stress decreases by approximately 0.015 MPa, and the maximum strain decreases by approximately 0.012 for each increase of 1 in the length-to-diameter ratio *n*. For path 2, the maximum stress decreases by approximately 0.010 MPa, and the maximum strain decreases by approximately 0.007 for each increase of 1 in the length-to-diameter ratio *n*.

## 5. Conclusions

This study numerically investigated residual stress and strain during the curing and molding of HTPB two-stage solid propellants. The formation mechanisms were formulated, the relaxation modulus was characterized using a Prony series and the WLF time–temperature superposition equation, the curing and cooling stages were simulated, and the effects of modulus *m* and length-to-diameter ratio *n* were evaluated. The results show that:

(1) During curing, the temperature and curing degree on the side of the grain close to the core mold are higher than those on the side close to the shell. By the end of curing, both temperature and curing degree become nearly uniform throughout the grain. At the end of the curing and cooling processes, there are high stress and strain zones on both sides of the grain, close to the core mold and the side close to the shell. Among them, the high stress zone on the side close to the core mold is on the second-stage grain with a larger modulus, and the high strain zone is on the first-stage grain with a smaller modulus, which will affect the deformation and cracking of the grain surface. The high stress and strain zones on the side close to the shell are at the connection between the grain ends and the insulation layer, which will affect the debonding between the grain and the insulation layer. At the connection between the first-stage and second-stage grain, due to the different materials, there is a sudden change in stress and strain, and the maximum stress and strain occur. The proportion of residual stress generated during the curing process is 32.1%, and the proportion of residual strain is 32.6%.
(2) At a constant length-to-diameter ratio *n*, as the modulus *m* increases, the temperature and curing degree at the end of the curing process of the grain increase, and the temperature and curing degree differences between the side close to the core mold and the side close to the shell also increase. The amplitude of the maximum temperature and the curing degree change gradually decrease. The stress and strain distributions at the end of the cooling process of the propellant grain with different moduli *m* are similar. As the modulus *m* increases, the overall stress and strain of the grain increase, and the amplitude of the maximum stress and strain change on the side close to the core mold is larger than that on the side close to the shell. On the side close to the core mold, for each increase of 1 in the modulus *m*, the maximum stress increases by approximately 0.028 MPa, and the maximum strain increases by approximately 0.021. On the side close to the shell, for each increase of 1 in the modulus *m*, the maximum stress increases by approximately 0.008 MPa, and the maximum strain increases by approximately 0.007.
(3) At a constant modulus *m*, as the length-to-diameter ratio *n* increases, the temperature and curing degree at the end of the curing process of the grain decrease, and the temperature and curing degree differences between the side of the grain close to the core mold and the side close to the shell tend to be consistent. The amplitude of the maximum temperature change gradually increases, whereas the amplitude of the maximum curing degree change gradually decreases. The stress and strain distribution at the end of the cooling process of the propellant grains with different length-to-diameter ratios *n* is similar. As the length-to-diameter ratio *n* increases, the overall stress and strain of the grain decrease. On the side close to the core mold, for each increase of 1 in the length-to-diameter ratio *n*, the maximum stress decreases by approximately 0.015 MPa, and the maximum strain decreases by approximately 0.012. On the side close to the shell, for each increase of 1 in the length-to-diameter ratio *n*, the maximum stress decreases by approximately 0.010 MPa, and the maximum strain decreases by approximately 0.007.

## Figures and Tables

**Table 1:** Verification of mesh independence.

Mesh Schemes	Number of Elements	Number of Nodes	Maximum Stress/MPa	Error/%
Fine mesh	81,000	90,145	0.115	
Medium mesh	38,720	44,836	0.112	3
Coarse mesh	18,976	22,840	0.105	9

**Table 2:** Material parameters.

Material Parameters	First-Stage Propellant	Second-Stage Propellant	Insulation Layer	Shell	Core Mold
Thermal conductivity/[W/(m·°C)]	0.43	0.46	0.19	29.33	162.18
Specific heat/[J/(kg·°C)]	1.15 × 10^3^	1.23 × 10^3^	1.41 × 10^3^	460.56	880.25
Density/(kg/m^3^)	1.71 × 10^3^	1.75 × 10^3^	1.16 × 10^3^	7.85 × 10^3^	2.78 × 10^3^
Coefficient of thermal Expansion/(1/°C)	8.37 × 10^−5^	9.32 × 10^−5^	3.53 × 10^−4^	1.11 × 10^−5^	23.12 × 10^−6^
Poisson’s ratio	0.491	0.493	0.490	0.282	0.324
Relaxation modulus/MPa	/	/	23.12	2.14 × 10^5^	71.49

**Table 3:** *N*-th order curing kinetics parameters.

Hα/(J/kg^−1^)	*A*/(s^−1^)	*n*	*E*/(J/mol^−1^)
253,220	5.66 × 10^7^	0.92	89,650

**Table 4:** Lg*α_T_*-*T* relationship.

***T*** **/°C**	70	50	20	0	−20	−40
**lg*α_T_***	−2.941	−1.955	0	1.082	2.884	6.317

**Table 5:** Prony series of the first-stage propellant.

***i***	1	2	3	4	5	6	7	8	9	10	11
***E_i_*** **/MPa**	0.678	0.652	0.645	0.459	0.331	0.327	0.280	0.244	0.189	0.170	0.153

**Table 6:** Prony series of the second-stage propellant.

***i***	1	2	3	4	5	6	7	8	9
***E_i_*** **/MPa**	1.117	1.005	0.387	0.348	0.317	0.286	0.261	0.235	0.214

## Data Availability

The original contributions presented in this study are included in the article. Further inquiries can be directed to the corresponding author.
